# Programming active cohesive granular matter with mechanically induced phase changes

**DOI:** 10.1126/sciadv.abe8494

**Published:** 2021-04-23

**Authors:** Shengkai Li, Bahnisikha Dutta, Sarah Cannon, Joshua J. Daymude, Ram Avinery, Enes Aydin, Andréa W. Richa, Daniel I. Goldman, Dana Randall

**Affiliations:** 1 School of Physics, Georgia Institute of Technology, Atlanta, GA 30332, USA.; 2 School of Electrical and Computer Engineering, Georgia Institute of Technology, Atlanta, GA 30332, USA.; 3 Mathematical Sciences, Claremont McKenna College, Claremont, CA 91711, USA.; 4 Computer Science, CIDSE, Arizona State University, Tempe, AZ 85281, USA.; 5 School of Computer Science, Georgia Institute of Technology, Atlanta, GA 30332, USA.; 6 Santa Fe Institute, 1399 Hyde Park Road, Santa Fe, NM 87501, USA.

## Abstract

At the macroscale, controlling robotic swarms typically uses substantial memory, processing power, and coordination unavailable at the microscale, e.g., for colloidal robots, which could be useful for fighting disease, fabricating intelligent textiles, and designing nanocomputers. To develop principles that can leverage physical interactions and thus be used across scales, we take a two-pronged approach: a theoretical abstraction of self-organizing particle systems and an experimental robot system of active cohesive granular matter that intentionally lacks digital electronic computation and communication, using minimal (or no) sensing and control. As predicted by theory, as interparticle attraction increases, the collective transitions from dispersed to a compact phase. When aggregated, the collective can transport non-robot “impurities,” thus performing an emergent task driven by the physics underlying the transition. These results reveal a fruitful interplay between algorithm design and active matter robophysics that can result in principles for programming collectives without the need for complex algorithms or capabilities.

## Teaser

Interactions of simple robots can be mapped to a discrete algorithm that accurately predicts the ensemble’s emergent behaviors.

## INTRODUCTION

Self-organizing collective behaviors are found throughout nature, including shoals of fish aggregating to intimidate predators (*1*), fire ants forming rafts to survive floods (*2*), and bacteria forming biofilms to share nutrients when they are metabolically stressed (*3*). Inspired by such systems, researchers in swarm robotics and programmable active matter have used many approaches toward enabling ensembles of simple, independent units to cooperatively accomplish complex tasks (*4*–*6*). Both control theoretic and distributed computing approaches have achieved some success, but often rely critically on robots computing and communicating complex state information, requiring relatively sophisticated hardware that can be prohibitive at small scales (*7*, *8*). Alternatively, statistical physics approaches model swarms as systems being driven away from thermal equilibrium by robot interactions and movements [see, e.g., (*9*, *10*)]. Tools from statistical physics such as the Langevin and Fokker-Planck equations can then be used to analyze the mesoscopic and macroscopic system behaviors (*11*). Current approaches present inherent trade-offs, especially as individual robots become smaller and have limited functional capabilities (*12*, *13*) or approach the thermodynamic limits of computing and power (*14*).

To apply to a general class of micro- or nanoscale devices with limited capabilities, we focus on systems of autonomous, self-actuated entities that use strictly local interactions to induce macroscale behaviors. Two behaviors of interest are dynamic free aggregation, where agents gather together without preference for a specific aggregation site [see section 3.2.1 of (*5*)], and dispersion, its inverse. These problems are widely studied, but most work either considers robots or models with relatively powerful capabilities—e.g., persistent memory for complex state information (*15*, *16*) or long-range communication and sensing (*17*–*19*)—or lacks rigorous mathematical foundations explaining the generality and limitations of their results as sizes scale (*20*–*22*). Recent studies on active interacting particles (*23*) and inertial, self-organizing robots (*24*) use physical models to treat aggregation and clustering behaviors, but neither prove behavior guarantees that scale with system size and volume. Supersmarticle ensembles (*25*) are substantially more complex, exhibiting many transient behavioral patterns stemming from their many degrees of freedom and chaotic interactions, making them less amenable to rigorous algorithmic analysis.

Here, we take a two-pronged approach to understanding the fundamental principles of programming task-oriented matter that can be implemented across scales without requiring sophisticated hardware or traditional computation that leverages the physics of local interactions. We use a theoretical abstraction of self-organizing particle systems (SOPS), where we can design and rigorously analyze simple distributed algorithms to accomplish specific goals that are flexible and robust to errors. We then build a new system of deliberately rudimentary active “cohesive granular robots” (which, to honor granular physics pioneer Robert Behringer, we call “BOBbots” for Behaving, Organizing, Buzzing robots) to test whether the theoretical predictions can be realized in a real-world damped driven system. The lattice-based equilibrium model quantitatively captures the aggregation dynamics of the robots. With a provable algorithmic model and even simpler BOBbots capturing the algorithm’s essential rules, we next explore how contact stress sensing—a capability that is readily available in the robotic platform but not easily computable by a strictly local, distributed algorithm—can enhance aggregation performance, as suggested by insights from the theoretical model. This complementary approach demonstrates a fruitful integration of the fields of distributed algorithms, active matter, and granular physics that navigates a translation from theoretical abstraction to practice, using methodologies inherent to each field.

## RESULTS

### Aggregation algorithm

While many systems use interparticle attraction and sterical exclusion to achieve system-wide aggregation and interparticle repulsion to achieve dispersion, these methods typically use some long-range sensing and tend to be nonrigorous, lacking formal proofs guaranteeing desirable system behavior. To better understand these collective behaviors, the abstract model of SOPS allows us to define a formal distributed algorithm and rigorously quantify long-term behavior. Particles in a SOPS exist on the nodes (or vertices) of a lattice, with at most one particle per node, and move between nodes along lattice edges. Each particle is anonymous (unlabeled), interacts only with particles occupying adjacent lattice nodes, and does not have access to any global information such as a coordinate system or the total number of particles.

In earlier work, Cannon *et al*. (*26*) analyzed a distributed SOPS algorithm for aggregation and dispersion under the assumption that the particle system remained simply connected (i.e., the system forms a single connected cluster with no holes). This SOPS algorithm defines a finite Markov chain with local moves that connect the state space of all simply connected configurations of particles. Moves are defined so that each particle, when activated by its own Poisson clock (i.e., after a delay chosen at random from a Poisson distribution with constant mean), chooses a random neighboring node and moves there with a probability that is a function of the number of neighbors in the current and new positions provided that the node is unoccupied and the move satisfies local conditions that guarantee that the configuration stays simply connected. In particular, for configurations σ and τ differing by the move of a single particle *p* along a lattice edge, the transition probability is defined as *P*(σ, τ) ∝ min (1, λ^*n*^′^−*n*^), where λ > 0 is a bias parameter that is an input to the algorithm, *n* is the number of neighbors of *p* in σ, and *n*′ is the number of neighbors of *p* in τ. These probabilities arise from the celebrated Metropolis-Hastings algorithm (*27*, *28*) and are defined so that the Markov chain converges to a unique Boltzmann distribution π such that π(σ) is proportional to λ^*E*(σ)^, where *E*(σ) is the number of nearest neighbor pairs in σ (i.e., those pairs that are adjacent on the lattice).

It was shown in (*26*) that the connected SOPS ensemble provably aggregates into a compact conformation when λ > 3.42 and expands to a conformation with nearly maximal (linear) perimeter when λ < 2.17 with high probability, i.e., with a probability of failure that is exponentially small in *N*, the number of particles. However, despite rigorously achieving both aggregation and dispersion, this distributed algorithm has two notable drawbacks that make it infeasible for direct implementation in a physical system of simple robots: the connectivity requirement that tethers the particles together and the “look ahead” requirement used to calculate transition probabilities ensuring convergence to the desired Boltzmann distribution.

**Fig. 1:**
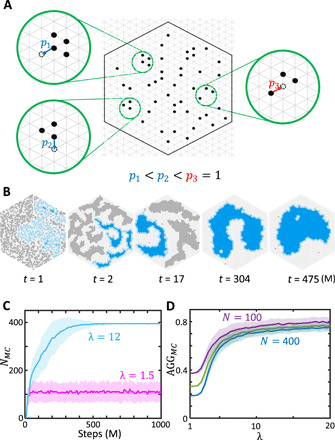
The self-organizing particle systems (SOPS). (**A**) A particle moves away from a node where it has *n* neighbors with probability λ^−*n*^, where λ > 0. Thus, moves from locations with more neighbors are made with smaller probability than those with fewer (e.g., in the insets, *p*_1_ = λ^−3^ < *p*_2_ = λ^−2^ < *p*_3_ = 1). (**B**) Time evolution of a simulated SOPS with 1377 particles for λ = 7.5 showing progressive aggregation (movie S1). The bulk of the largest connected component is shown in blue, and its periphery is shown in light blue. (**C**) Time evolution of *N_MC_*, the size of the largest connected component, showing dispersion for λ = 1.5 and aggregation for λ = 12. The simulations use 400 particles. (**D**) Phase change in λ space for the aggregation metric $\text{AG}G_{\mathrm{MC}}=N_{\mathrm{MC}}/(k_{0}P_{\mathrm{MC}}\sqrt{N})$, where *k*_0_ is a scaling constant, *P_MC_* is the number of particles on the periphery of the largest component, and *N* is the total number of particles. This phase change is qualitatively invariant to the system’s size.

To address these issues, we define a modified aggregation and dispersion algorithm M_AGG_ where particles can disconnect and moves rely only on the current state. Here, particles occupy nodes of a finite region of the triangular lattice, again moving stochastically and favoring configurations with more pairs of neighboring particles. Each particle has its own Poisson clock and, when activated, chooses a random adjacent lattice node. If that node is unoccupied, then the particle moves there with probability λ^−*n*^, where *n* is the number of current neighbors of the particle, for bias parameter λ > 0. Thus, rather than biasing particles toward nodes with more neighbors, we instead discourage moves away from nodes with more neighbors, with larger λ corresponding to a stronger ferromagnetic attraction between particles (Fig. 1A). This new chain M_AGG_ converges to the same Boltzmann distribution π(σ) ∝ λ^*E*(σ)^ over particle system configurations σ as the original SOPS algorithm. Details of the proofs can be found in Materials and Methods.

Let Ω be the set of configurations with *N* particles within our bounded lattice region. We will use the following definition to quantify aggregation for particles that can be disconnected, capturing both the size and compactness of aggregates.

**Definition 1.** For β > 0 and δ ∈ (0,1/2), a configuration σ ∈ Ω is (β, δ) aggregated if there is a subset *R* of lattice nodes such that 1)At most $\beta \sqrt{N}$ edges have exactly one end point in *R*; 2)The density of particles in *R* is at least 1 − δ; and 3)The density of particles not in *R* is at most δ.

Here, β is a measure of how small the boundary between *R* and its complement $\bar{R}$ must be, measuring the compactness of the aggregated particles, and δ is a tolerance for having unoccupied nodes within the cluster *R* or occupied nodes outside of *R*. We say that a configuration is dispersed if no such (β, δ) exist.

By carefully analyzing the stationary distribution of ℳ_AGG_, which is the desired Boltzmann distribution, we establish conditions that provably yield aggregation when the particles are confined to a compact region of the triangular lattice (Fig. 1B). The proof uses arguments from Cannon *et al*. (*29*); see Materials and Methods for details.

**Theorem 2.** Let configuration σ be drawn from the stationary distribution of ℳ_AGG_ on a bounded, compact region of the triangular lattice, when the number of particles N is sufficiently large. If λ > 5.66, then with high probability, there exist β > 0 and 0 < δ < 1/2 such that σ will be (β, δ) aggregated. However, when 0.98 < λ < 1.02, the configuration σ will be dispersed with high probability.

Varying values of λ in simulation gives strong indication that dispersion persists for larger values of λ, and the aggregation algorithm undergoes a phase transition whereby the macroscopic behavior of the system suddenly changes from dispersion to aggregation (Fig. 1, C and D, and movie S1), mimicking the fixed magnetization ferromagnetic Ising model, which motivated our Markov chain algorithm. Nonetheless, our proofs demonstrate that our system has two distinct phases of behavior for different ranges of λ for a sufficiently large number of interacting particles, which is enough for our purposes.

### BOBbots: A model active cohesive granular matter system

**Fig. 2:**
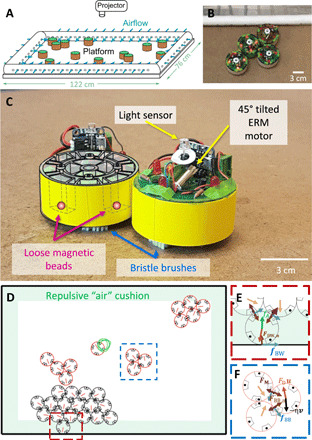
BOBbots and their collective motion. (**A**) Schematic of experimental setup. BOBbots are placed in a level arena with airflow gently repelling them from the boundaries. (**B**) A close-up of the experimental platform. (**C**) Mechanics of the BOBbots. Loose magnetic beads housed in the BOBbots’ peripheries can reorient so that BOBbots always attract each other. The vibration of the ERM motor and the asymmetry of bristles lead to the directed motion. The light sensor activates the motion. (**D**) DEM simulation setup. (**E**) BOBbot-boundary interactions: airflow repulsion *f_A_*, BOBbot-boundary friction *f*_BW_, and normal force *F*_BW, n_. (**F**) Inter-BOBbot interactions: attraction between magnetic beads *F_M_*, inter-BOBbot friction *f*_BB_, and sterical exclusion *F*_BB, n_. Photo credit: Bahnisikha Dutta and Ram Avinery, Georgia Institute of Technology.

Next, to test whether the lattice-based equilibrium system can be used to control a real-world swarm in which there are no guarantees of detailed balance or Boltzmann distributions, we introduce a collective of active cohesive granular robots that we name BOBbots (Fig. 2, A to C, and fig. S1)—Behaving, Organizing, Buzzing robots—whose design physically embodies the aggregation algorithm. Driven granular media provide a useful soft matter system to integrate features of the physical world into the toolkit for programming collectives. This builds upon three decades of work understanding how forced collections of simple particles interacting locally can lead to remarkably complex and diverse phenomena, not only mimicking solids, fluids, and gasses (*30*–*32*)—e.g., in pattern formation (*33*, *34*), supercooled and glassy phenomena (*35*, *36*), and shock waves (*37*)—but also displaying phenomena characteristic of soft matter systems such as stress chains (*38*) and jamming transitions (*39*, *40*). While cohesive granular materials are typically generated in situations where particles are small (powders, with interactions dominated by electrostatic or even van der Waals interactions) or wet (with interactions dominated by formation of liquid bridges between particles) (*41*, *42*), we generate our cohesivity using magnets.

The movement and interactions between BOBbots were designed to capture the salient features of the abstract stochastic algorithm while replacing all sensing, communication, and probabilistic computation with physical morphology and interactions. Each BOBbot has a cylindrical chassis with a base of elastic “brushes” that are physically coupled to an off-center eccentric rotating mass vibration motor (ERM). The vibrations caused by the rotation of the ERM are converted into locomotion by the brushes (Fig. 2C). Because of asymmetry in our construction of this propulsion mechanism, the BOBbots traverse predominantly circular trajectories (*43*) that are randomized through their initial conditions but, unlike the SOPS particles, are inherently deterministic with some noise and occur at a constant speed per robot distributed as *v*_0_ = 4.8 ± 2.0 cm/s. See Materials and Methods for further details.

Analogous to the modified transition probabilities in the aggregation algorithm that discourage particles from moving away from positions where they have many neighbors, each BOBbot has loose magnets housed in shells around its periphery that always reorient to be attractive to nearby BOBbots (Fig. 2C). The probability that a BOBbot detaches from its neighbors is negatively correlated with the attractive force from the number of engaged magnets, approximating the movement probabilities given by the algorithm that scale inversely and geometrically with the number of neighbors. We subsequently verify this assertion experimentally (see section S5 for details). The strength of the magnets *F*_*M*0_ determines whether the system aggregates or disperses in the long run, analogous to λ in the algorithm.

To allow for study of larger BOBbot ensembles and more comprehensive sweeps of parameter space, we also performed discrete element method (DEM) simulations of the BOBbots (see Fig. 2, D to F, and Materials and Methods for more details). The motion of an individual BOBbot is modeled as a set of overdamped Langevin-type equations governing both its translation and rotation subject to its diffusion, drift (*44*), magnetic attraction, and sterical exclusion with other BOBbots. The translational drift corresponds to the speed from the equilibrium of the drive and drag forces, while the rotational drift corresponds to the circular rotation. Similar methods have been used to understand macroscale phenomena emerging from collectives of microscopic elements (*11*) and to model particle motion in active matter (*45*).

Mitigating the effects of the arena’s fixed boundaries in both experiments and simulations presented a design challenge. BOBbots can persist along the boundary or in corners, affecting system dynamics by, for example, enabling aggregates to form where they would not have otherwise or hindering multiple aggregates from integrating. To address these issues, uniform airflow was used to gently repel BOBbots away from the boundary, and similar effects were implemented in simulation. More details about the experimental apparatus and protocol can be found in Materials and Methods.

### Clustering dynamics explained by algorithm analysis

**Fig. 3:**
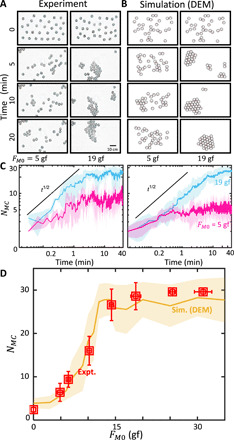
Evolution of BOBbot clusters. (**A**) Time evolution snapshots of both experiment (movie S3) and (**B**) simulation (movie S4) for a system of 30 BOBbots with different magnet strengths: *F*_*M*0_ = 5 gf (left) where we observe dispersion, and *F*_*M*0_ = 19 gf (right) where we observe aggregation. Experimental images have been processed with a low-pass filter for better visual clarity. (**C**) Time evolutions of the size of the largest component *N_MC_* in experiment and simulation for a system of 30 BOBbots with *F*_*M*0_ = 5 gf (magenta) and *F*_*M*0_ = 19 gf (blue). (**D**) Scaling of cluster size versus magnetic strength for a system of 30 BOBbots showing an increase in *N_MC_* as the magnet strength *F*_*M*0_ increases. The yellow plot line shows the mean and SD of *N_MC_* in the 150 simulation runs for each magnetic strength *F*_*M*0_ between 1 and 35 gf, with a step size of 1 gf. Experimental data are shown in red, with error bars showing the SD of the largest cluster size *N_MC_* and the uncertainty of *F*_*M*0_ due to empirical measurement.

Since the critical elements of the SOPS algorithm can be physically embodied by robots as simple as our BOBbots, to test whether the SOPS model could quantitatively capture collective dynamics, we next investigated the degree to which collectives of BOBbots aggregate as a function of their peripheral magnet strength *F*_*M*0_ in both robotic experiments and DEM simulations. (For convenience, we use gram-force (gf) as the unit for the magnetic force where 1 gf = 1 gram × 9.81 m/s^2^ when using the unit of gram.) The experimental protocol begins with placing magnets of a particular strength *F*_*M*0_ into the BOBbots’ peripheral slots. The BOBbots are positioned and oriented randomly in a rectangular arena and are then actuated uniformly for a fixed time during which the BOBbots’ positions and the size of the largest connected component are tracked (Fig. 3, A to C). These trials are conducted for several *F*_*M*0_ values with repetition. We followed the same protocol in simulations.

In experiment and DEM simulation, we observe an abrupt, rapid rise and then saturation in the size *N_MC_* of the largest connected component as the magnetic attraction *F*_*M*0_ increases (Fig. 3D). These curves resemble those in Fig. 1D, with the magnetization *F*_*M*0_ playing a role analogous to the bias parameter λ. Given this correspondence, we explored whether the equilibrium SOPS model could be used to make quantifiable predictions in the robot experiments. First, we designed a test to examine how force and λ scale. Recall that in the SOPS algorithm, the force acting on each particle is proportional to λ*^n^*, where *n* is the particle’s current number of neighbors. In the experiments, BOBbots cannot count their neighbors, but the magnets are expected to provide a similar force that also increases geometrically when more magnets are engaged.

**Fig. 4:**
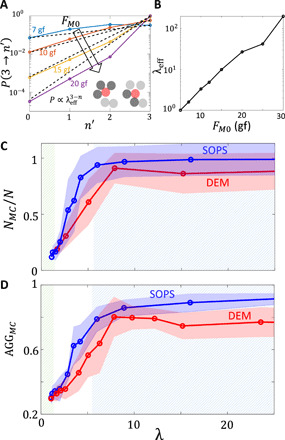
Algorithmic interpretation of BOBbot clustering. (**A**) Diagram showing how the effective bias parameter λ_eff_ is evaluated from the DEM simulation. (**B**) Dependence of λ_eff_ on the magnetic attraction force *F*_*M*0_. (**C**) Maximum cluster fraction *N_MC_*/*N* and (**D**) aggregation metric AGG*_MC_* for different values of λ in both the SOPS algorithm (blue) and physical simulations (red). The green and blue shaded regions show the dispersed and aggregated regimes proved from theory, respectively.

To estimate the relationship between force and λ, we investigate the rate at which a BOBbot loses or gains neighbors over a fixed amount of time. Viewing a BOBbot’s completion of half its circular motion as analogous to a particle moving to a new lattice node in the SOPS algorithm and using this time interval to evaluate the transition, simulation data show that a BOBbot’s transition probability from having a higher number of neighbors *n* to a lower number *n*′ closely follows the algorithm’s *P*(σ, τ) ∝ min (1, λ^*n*^′^−*n*^) transition probabilities (Fig. 4A and fig. S10). Further, we evaluated the BOBbots’ effective bias parameter λ_eff_ as a function of *F*_*M*0_ and found an exponential relation λ_eff_ = exp (β*F*_*M*0_), where β is a constant representing inverse temperature (Fig. 4B). The BOBbots’ transition probabilities can then be approximated as *P*(σ, τ) = exp(− β(ϵ*_n_* − ϵ_*n*^′^_)), where β is the inverse temperature of the system and ϵ*_n_* = *n* · *F*_*M*0_ can be interpreted as the energy contributed by a BOBbot’s *n* neighbors.

With the relation between *F*_*M*0_ and λ_eff_ established, we next compare the aggregation behaviors exhibited by the SOPS algorithm and the BOBbot ensembles. Figure 4C shows the fraction of particles/BOBbots in the largest component *N_MC_*/*N* observed in both the SOPS algorithm and BOBbot simulations after converting with respect to λ_eff_; the algorithm does capture the maximum cluster fraction observed in the simulations. Notably, the aggregated and dispersed regimes in λ space established in Theorem 2 provide a rigorous understanding of these BOBbot collective behaviors. For instance, the proven dispersed regime 0.98 < λ < 1.02 gives a clear explanation for why agents will not aggregate even in the presence of mutual attraction. Further, it also helps establish the magnitude of attraction needed to saturate the aggregation.

**Fig. 5:**
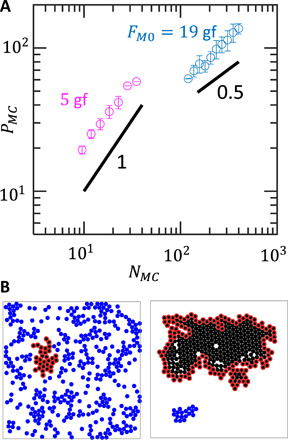
Perimeter scaling of BOBbot clusters. (**A**) Log-log plot showing the scaling relationship between the largest component’s size *N_MC_* and perimeter *P_MC_* in number of BOBbots for simulated systems of 400 BOBbots with *F*_*M*0_ = 5 gf (magenta) and 19 gf (cyan) for fixed boundary conditions. Each data point is the average of 20 simulations. While the SOPS predicts a scaling power of 0.5 for the aggregated case (cyan), the data show a slightly larger, but still sublinear, power of 0.66 ± 0.07. (**B**) Final snapshot of the collective motion of 400 BOBbots with *F*_*M*0_ = 5 gf (left) and 19 gf (right). BOBbots shown in black belong to the largest connected component; those outlined in red are on its periphery.

We additionally test the SOPS prediction that the maximum cluster should not only be large but also compact, occupying a densely packed region. The results from Cannon *et al*. (*29*) that we apply here for aggregation suggest the following relationship between the size of the largest component *N_MC_* and its perimeter *P_MC_*. In dispersed configurations, *P_MC_* should scale linearly with *N_MC_*, meaning that most BOBbots lie on the periphery of their components. In aggregated configurations, however, *P_MC_* should scale as $N_{\mathrm{MC}}^{1/2}$, approximating the minimal perimeter for the same number of BOBbots by at most a constant factor. We test these scaling relationships in simulations with 400 BOBbots (Fig. 5A) and find that the theory’s predictions hold in the dispersed regime; however, the 0.66 ± 0.07 sublinear scaling power for the aggregated case is slightly higher than the theory’s prediction of 0.5. This discrepancy may, in part, be due to boundary and finite-size effects—in fact, DEM simulations with periodic boundaries show a scaling power of 0.59 ± 0.18 that is closer to the SOPS theory (fig. S17)—but is also affected by nonreversibility inherent in the BOBbots’ circular trajectories. To make quantitative comparison that captures when components are both large and compact, we track ${\text{AGG}}_{\mathrm{MC}}=N_{\mathrm{MC}}/(k_{0}P_{\mathrm{MC}}\sqrt{N})$, where *k*_0_ is a scaling constant defined such that AGG*_MC_* = 1 when the system is optimally aggregated, achieving the minimum possible perimeter. Physically, AGG*_MC_* is reminiscent of the surface tension for which energy minimization leads to a smaller interface (in our setting, smaller perimeter *P_MC_*), yielding an AGG*_MC_* closer to 1. We obtain agreement between the SOPS and DEM simulations with respect to this metric as well (Fig. 4D), further validating the theory’s prediction, although the DEM simulations yield slightly smaller AGG*_MC_* than the SOPS algorithm for large λ.

We noticed that the size of the largest component *N_MC_* grows roughly proportional to *t*^1/2^ over time (Fig. 3C). Since the perimeter of the largest cluster *P_MC_* scales proportional to $N_{\mathrm{MC}}^{0.66}\approx N_{\mathrm{MC}}^{2/3}$ (Fig. 5A), this implies that the length scale grows like *t*^1/3^. This is reminiscent of coarsening in a broad class of systems described by the Cahn-Hilliard equation ∂*u*/*∂t* = ∇^2^(Φ′(*u*) − γ∇^2^*u*), where order parameter *u* takes continuous values in (−1,1), where −1 and 1 are analogous to empty and occupied nodes in the SOPS lattice, respectively. To bridge the SOPS algorithm with the Cahn-Hilliard equation, we first observe that the SOPS algorithm with bias parameter λ can be exactly mapped to an Ising model with fixed magnetization (*46*, *47*) with coupling strength $J=\frac{1}{2\beta }\text{log}\;\lambda$, where β is the inverse temperature (see section S7 for details). As shown by Penrose (*48*), the fixed magnetization Ising model with coupling strength *J* can be mapped to the surface tension γ of the Cahn-Hilliard equation as γ = β*J*. Thus, the SOPS and BOBbot ensemble behaviors map to the Cahn-Hilliard equation with $\gamma =\frac{1}{2}\text{log}\;\lambda \propto F_{M0}$. This suggests that, in the limit, the SOPS and BOBbot aggregation behavior should display a second-order phase transition at a critical λ*_c_* corresponding to the critical surface tension γ in the Cahn-Hilliard equation. The corresponding critical value λ*_c_* = *e*^2/7^ ≈ 1.33 on the hexagonal lattice lies within the λ*_c_* ∈ (1.02,5.66) range proven by the SOPS theory (see section S8 for details). Thus, we obtain agreement between the SOPS theory for a finite lattice system and the Cahn-Hilliard equation for an active matter system at the continuum limit. This mapping gives further confirmation of the universality of our results and provides another perspective for “programming” active collectives.

### Enhancing clustering via local stress sensing

We have demonstrated that the BOBbot ensembles mimic a lattice model that can provably aggregate for large enough λ, corresponding physically to highly attractive interaction that favors large components with small perimeter. We now ask whether we can achieve rudimentary collective intelligence determining, for example, how robots could tune their responses to enhance or dampen aggregation, thereby achieving a more tightly clustered or dispersed state. In particular, we explore whether such tuning can help counteract some ways the system deviates from the theory, such as variations in the BOBbots’ speeds and magnetic attraction, improving the fidelity to the original algorithm. While the BOBbots remain unable to count neighbors or estimate the Gibbs probabilities directly as prescribed by the algorithm, we take advantage of physical effects of the BOBbot ensembles to “program” desirable behavior without using any traditional computation.

**Fig. 6:**
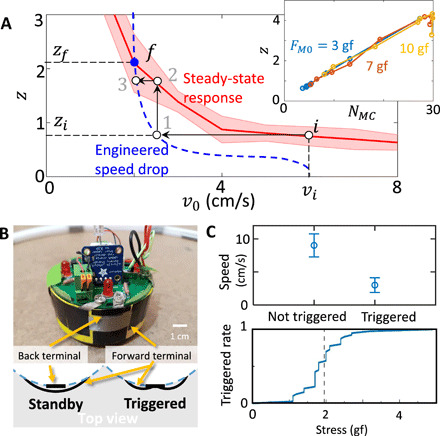
Design and implementation of stress sensing for enhanced aggregation. (**A**) Effect of the engineered, adaptive speeds (blue) on the steady-state average number of neighbors per BOBbot (red) for *F*_*M*0_ = 3 gf. Without adapting speeds, BOBbots actuated at a given speed *v_i_* would obtain an average of *z_i_* neighbors per BOBbot at equilibrium (initial point *i*). With the adaptive speeds, an average of *z_i_* neighbors per BOBbot causes the average speed to slow (*i* → 1), which, in turn, enables convergence to the steady-state response with more neighbors per BOBbot (1 → 2). This feedback iterates until the steady-state and engineered responses coincide at final point *f* = (*v_f_*, *z_f_*), where *v_f_* < *v_i_* and *z_f_* > *z_i_*. Inset: The mapping between the maximum cluster size *N_MC_* and the average number of neighbors per BOBbot *z* indicates that the stress-sensing control strategy will increase component sizes. (**B**) BOBbot equipped with a stress sensor and schematic top-view sketch of the triggered and not triggered states. (**C**) BOBbot’s response to stress. Top: Speed of a BOBbot when its sensor is and is not triggered. Bottom: Rate of sensor triggering as a function of the stress applied. Photo credit: Ram Avinery, Georgia Institute of Technology.

The first effect relies on observations that for a fixed magnet strength, the size of the largest component *N_MC_* decreases with increasing BOBbot speed *v*_0_ (fig. S9); a full investigation of the behavior of BOBbot collectives at varying uniform speeds will be the subject of a separate study. We further observe that *N_MC_* scales linearly with *z*, the average number of neighbors per BOBbot at equilibrium (Fig. 6A, inset). Thus, BOBbot speed *v*_0_ is inversely correlated with the average number of neighbors per BOBbot *z*. This arises from *v*_0_ being a proxy for β^−1^ in the effective attraction λ_eff_. Consequently, we can mimic enhanced aggregation via increased magnet strength by reducing a BOBbot’s speed as a function of its number of neighbors.

Without adapting a BOBbot’s speed based on its number of neighbors, a BOBbot collective actuated uniformly at a speed *v* converges to an average of *z*_std_(*v*) neighbors per BOBbot at equilibrium (Fig. 6A, red); any point in speed-neighbor space deviating from *z*_std_(*v*) is transient. To enhance aggregation, we engineer reduced speeds *v*_eng_(*z*) that a BOBbot with *z* neighbors should adapt to (Fig. 6A, blue). These slowed speeds allow the collective to reconverge to a new steady state with a larger number of average neighbors per BOBbot (Fig. 6A, arrows). This feedback between the engineered speeds *v*_eng_ and the steady-state average number of neighbors *z*_std_ iterates until reaching the fixed point in speed-neighbor space where the steady-state and engineered behaviors meet as *z* = *z*_std_(*v*_eng_(*z*)).

While adapting speeds based on numbers of neighbors would be relatively straightforward to implement in more complex robots capable of counting neighbors [e.g., optically as in (*15*, *16*, *49*, *50*)], implementing such a scheme in the deliberately simple BOBbots is challenging given their lack of such sensing. Here, we use a second physical effect: Inspired by the correlation of particle density and stress on individual particles in granular systems (*51*), we propose that monitoring local contact stress can function as a proxy for counting numbers of neighbors. An immediate benefit of such a scheme is that it can be implemented on the existing robots via custom, low-cost, analog surface stress sensors (see Fig. 6B and Materials and Methods for details). The implemented stress sensors function such that for sufficiently large stress (e.g., when in a cluster), motor speed is decreased by 70% (Fig. 6C).

**Fig. 7:**
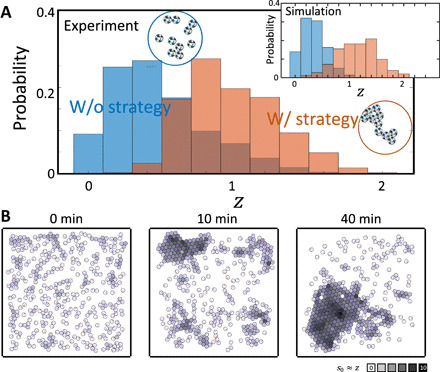
Adapting speed via stress sensing enhances aggregation. (**A**) Distribution of a BOBbot’s number of contacts over six 10-min experiments using *F*_*M*0_ = 3 gf. Each sample is an average of number of contacts over 1 s. Inset: Simulation results using the same conditions as the experiment. (**B**) A simulation demonstrating enhanced aggregation in an ensemble of 400 BOBbots using a weak magnet strength of *F*_*M*0_ = 7 gf. Each BOBbot’s speed decreases from 6 to 1.2 cm/s, as its stress *s*_0_ = Σ_*j*ϵneighbors_ *s_j_*/*F*_*M*0_ ≈ *z* increases from 0 to 6, where *z* is its current number of neighbors. BOBbots in an aggregate’s interior experience the most stress (dark gray) and thus have the slowest speeds, enabling larger aggregates to form. Without adapting speed in response to stress, the cluster sizes retain the same magnitude as in the 0-min snapshot (left).

We implemented this “physical algorithm” on BOBbot ensembles with weakly attractive magnets (movie S6). In experiments with ensembles of 10 BOBbots in a circular arena, adapting BOBbot speeds in response to stress sensing increases the average number of neighbors per BOBbot (Fig. 7A). Further, there is a quantitative match in the final average number of neighbors per BOBbot between the experiments and the fixed points predicted in Fig. 6A, validating our control strategy for enhancing aggregation. Simulations using the same arena and stress-mediated response reproduce the experimental results (Fig. 7A, inset). In simulations of 400 BOBbots with *F*_*M*0_ = 7 gf, we observe that BOBbots with more neighbors experience higher stress and thus have the slower speeds (Fig. 7B). This stress-mediated decrease in speed enables large aggregates to form that would not have existed otherwise in the weakly attractive regime. The use of stress sensing opens an interesting avenue for collectives of rudimentary robots to incorporate higher-order information without complex vision systems; further, contact stress provides insights (e.g., closeness to a jamming transition) that could be valuable in densely packed clusters (*52*).

### Object transport in the aggregated phase

Encouraged by the close connections between the physical system and the underlying theoretical model along with the successful control scheme for enhanced aggregation using stress sensing, we sought to test whether aggregated BOBbots could collectively accomplish a task. In particular, could an aggregated BOBbot collectively “recognize” the presence of a nonrobot impurity in its environment and cooperatively expel it from the system? Typically, such collective transport tasks—e.g., the cooperative transport of food by ants (*53*, *54*)—either manifest from an order-disorder transition or rely heavily on conformism between agents for concerted effort and alignment of forces. With our BOBbot collectives, we instead aim to accomplish transport via mechanics and physical interactions emergently controlling global behavior without complex control, communication, or computation.

**Fig. 8:**
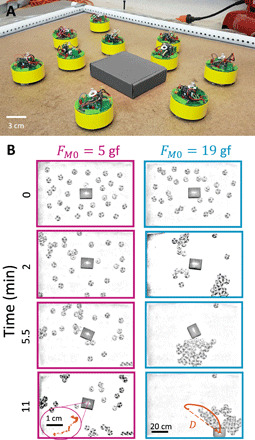
Object transport using aggregation. (**A**) Schematic of the experimental setup. (**B**) Time evolution snapshots of box transport by a system of 30 BOBbots with magnet strength *F*_*M*0_ = 5 and 19 gf (movie S7). The box has a mass of 60 g. The final panel shows the object’s complete trajectory, where *D* denotes the Euclidean distance of the final displacement. Photo credit: Ram Avinery and Bahnisikha Dutta, Georgia Institute of Technology.

By maintaining a high magnetic attraction *F*_*M*0_, we remain in the aggregated regime where most BOBbots connect physically and can cumulatively push against untethered impurities (e.g., a box or disk) introduced in the system (Fig. 8A and movie S7). The BOBbot collective’s constant stochastic reconfiguration grants it the ability to envelop, grasp, and dislodge impurities, as their individual forces additively overcome the impurities’ friction, leading to large displacement in the aggregated regime (Fig. 8B, right) with a median displacement of 7.9 cm over 12 min. On the contrary, we find that systems with weak magnetic attraction (i.e., those in the dispersed regime) can typically only achieve small impurity displacement (Fig. 8B, left) with a median displacement of 0.9 cm over 12 min (see fig. S11 for distributions). We observe infrequent anomalies in which dispersed collectives achieve larger displacement than aggregated ones, but these outliers arise from idiosyncrasies of our rudimentary robots (e.g., an aggregated cluster of BOBbots may continuously rotate in place without coming in contact with an impurity due to the BOBbots’ individual orientations in the aggregate; see movie S7).

**Fig. 9:**
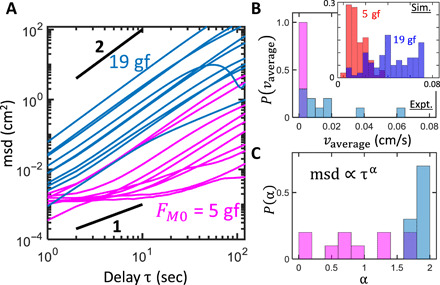
Characterizing the transport. (**A**) Mean squared displacement of the box over time in log-log scale for collectives with *F*_*M*0_ = 5 gf (magenta) and 19 gf (blue). (**B**) Distribution of the average speed, calculated as the final displacement *D* (as shown in Fig. 7B) divided by total time. Inset: Simulation results for the overall transport speed. Inset: Simulation results for the overall transport speed. The two peaks for *F*_*M*0_ = 19 gf correspond to pushing to the edges and corners. (**C**) Distributions of the mean squared displacement exponent α at short time scale τ < 20 s.

Characterizing the impurity’s transport dynamics as mean squared displacement over time <*r*^2^(τ) > = *v*τ^α^ reveals further disparities between the aggregated and dispersed BOBbot collectives (Fig. 9A). On a log-log plot, the intercept indicates log (*v*), where *v* is the characteristic speed of the impurity’s transport; we observe that, in all but one fringe case, the strongly attractive collectives achieve transport that is orders of magnitude faster than those of the weakly attractive ones (Fig. 9B). The slope of each trajectory indicates the exponent α that characterizes transport as subdiffusive (α < 1), diffusive (α = 1), or superdiffusive (α > 1). While all the strongly attractive collectives immediately achieve nearly ballistic transport (with α = 1.85 ± 0.11 for τ < 20 s), indicating rapid onset of cluster formation and pushing, the weakly attractive collectives initially exhibit mostly subdiffusive transport (with α = 0.89 ± 0.56 for τ < 20 s) caused by intermittent collisions from the dispersed BOBbots (Fig. 9C). When the slight heterogeneous distribution of the dispersed BOBbots remains unchanged for a sufficiently long time, the accumulation of displacement in a persistent direction can cause a small drift, leading to ballistic transport at a longer time scale. These results are in accord with the predictions of a simple model combining subdiffusive motion with small drift (fig. S12). Nonetheless, the transport speeds achieved by the dispersed collectives are two orders of magnitude smaller than those of the strongly attractive ones.

Simulations of impurity transport (see section S6 for details) reproduce the experimental results (Fig. 9B, inset, and movie S7), including the rare anomalies. Seven of the 100 simulations of weakly attractive collectives succeeded in transporting the impurity to the arena boundary at slow speeds, while 76 of the 100 simulations of strongly attractive collectives did so ballistically. The remaining 24 simulations of attractive collectives that did not achieve ballistic transport consistently formed an aggregate that never came into contact with the impurity. We found that disaggregating established aggregates by introducing time periods with no attraction enabled them to dissolve and reform for another attempt at transport. Using different disaggregating sequences, the attractive collectives achieved ballistic transport in 15 to 20% more simulations than without disaggregating (fig. S13). Physically and interestingly, in the Cahn-Hilliard picture, impurity transport can be interpreted as the expulsion of an obstacle in a continuum mixture with sufficiently high surface tension to yield phase separation. If the obstacle occupies a position that is later occupied by the solid phase, then the obstacle is expelled because of sterical exclusion; when its position is unvisited by the solid phase during the process of coarsening, however, it remains stagnant, similar to the anomalies for attractive collectives. In this interpretation, disaggregating effectively repeats the coarsening process to that the probability any given position is unvisited by the solid phase is diminished.

## DISCUSSION

In this paper, we use mathematical ideas from distributed computing and statistical physics to create task-oriented cohesive granular media composed of simple interacting robots called BOBbots. As predicted by the theory, the BOBbots aggregate compactly with stronger magnets (corresponding to large bias parameter λ) and disperse with weaker magnets (or small λ). Simulations capturing the physics governing the BOBbots’ motions and interactions further confirm the predicted phase change with larger numbers of BOBbots. The collective transport task then demonstrates the utility of the aggregation algorithm.

There are several noteworthy aspects of these findings. First, the theoretical framework of the underlying SOPS model can be generalized to allow many types of relaxations to its assumptions, provided that its dynamics remain reversible and model a system at thermal equilibrium. For example, noting that the probability that a robot with *n* neighbors detaches may not scale precisely as λ^−*n*^ as suggested by the Boltzmann weights, we can generalize the SOPS model to be more sensitive to small variations in these weights: The proofs establishing the two distinct phases can be shown to extend to this setting, provided the probabilities *p_n_* of detaching from *n* neighbors satisfy *c*_1_λ^−*n*^ ≤ *p_n_* ≤ *c*_2_λ^−*n*^, for constants *c*_1_, *c*_2_ > 0.

The robustness of the local, stochastic algorithms makes the macroscale behavior of the collective resistant to many types of idiosyncrasies inherent in the BOBbots, including bias in the directions of their movements, the continuous nature of their trajectories, and nonuniformity in their speeds and magnet strengths. Moreover, our algorithms are inherently self-stabilizing due to their memoryless, stateless nature, always converging to a desired system configuration overcoming faults and other perturbations in the system without the need for external intervention. In our context, the algorithm will naturally continue to aggregate, even as some robots may fail or the environment is perturbed.

We find agreement not only between the BOBbot ensembles and the discrete SOPS model but also with continuum models. The SOPS algorithm for aggregation and dispersion was initially defined as a distributed, stochastic implementation of a fixed magnetization Ising model. In addition to showing that our experimental system follows guarantees established by the analysis of a discrete model, we also observe that the growth of its largest component matches the power law derived for the Cahn-Hilliard equation, a continuous analog of the Ising model (*48*). This mapping provides an intuitive understanding of how the SOPS bias parameter λ, the physical inter-BOBbot attraction *F*_*M*0_, and the surface tension γ in the Cahn-Hilliard equation correspond; thus, as γ controls the phase change in the Cahn-Hilliard equation, so do λ and *F*_*M*0_ in their respective settings. This observation buttresses our confidence that the SOPS model provides a useful algorithmic framework capable of producing valid statistical guarantees for ensembles of interacting robots in continuous space.

We find that the nonequilibrium dynamics of the BOBbots are largely captured by the theoretical models that we analyze at thermal equilibrium, which is in agreement with the findings of Stenhammar *et al*. (*55*). For example, in addition to visually observing the phase change as the magnetic strengths increase, we are able to test precise predictions about the size and perimeter of the largest connected components based on the formal definitions of aggregation and dispersion from the SOPS model. We additionally use simulations to study the transition probability of a BOBbot from having *n* neighbors to having *n*′ neighbors to see whether the magnetic interactions conform to the theory, and we see a geometric relation decrease in the probability of moving as we increase the number of neighbors, as predicted. The resultant correspondence between the magnetic attraction and effective bias in the algorithm confirms a quantitative connection between the physical world and the abstract algorithm.

In summary, the framework presented here using provable distributed, stochastic algorithms to inspire the design of robust, simple systems of robots with limited computational capabilities seems quite general. It also allows one to leverage the extensive amount of work on distributed and stochastic algorithms and equilibrium models and proofs in guiding the tasks of inherently out-of-equilibrium robot swarms. Preliminary results show that we likely can achieve other basic tasks such as alignment, separation (or speciation), and flocking through a similar principled approach. We note that exploiting physical embodiment with minimal computation seems a critical step in scaling collective behavior to encompass many cutting-edge settings, including microsized devices that can be used in medical applications and cheap, scalable devices for space and terrestrial exploration. In addition, we plan to further study the important interplay between equilibrium and nonequilibrium dynamics to better solidify these connections and to understand which relaxations remain in the same universality classes.

## MATERIALS AND METHODS

### Details of the SOPS algorithm and proofs

The SOPS algorithm ℳ_AGG_ for aggregation and dispersion is given in Algorithm 1. The algorithm is presented as a Markov chain but could easily be modified to function as a distributed algorithm executed by each particle independently and concurrently, as shown in (*26*, *29*).

**Algorithm 1:** Markov chain ℳ_AGG_ for aggregation and dispersion in SOPS

Beginning at any configuration of *N* particles in a bounded region, fix λ > 1 and repeat: 1: Choose a particle *P* uniformly at random; let ℓ be the lattice node it occupies 2: Choose an adjacent lattice node ℓ′ and *q* ∈ (0,1) each uniformly at random. 3: **if** ℓ′ is empty and *q* < λ^−*n*^, where *n* is the number of neighbors *P* has at ℓ **then** 4: *P* moves to ℓ′. 5: **else** *P* remains at ℓ.

Recall that Theorem 2 analyzes the stationary distribution π of the Markov chain ℳ_AGG_ for aggregation and dispersion. In particular, Theorem 2 was shown in (*29*) to hold for π(σ) ∝ λ^−*b*(σ)^ ∝ λ^*E*(σ)^, where *b*(σ) is the number of “boundary edges” of the lattice that have exactly one end point occupied by a particle. So, it remains to show that ℳ_AGG_ converges to this stationary distribution π.

**Lemma 3.** The unique stationary distribution of ℳ_AGG_ is π(σ) = λ^−*b*(σ)^/*Z*, where *Z* = ∑_τ_λ^−*b*(τ)^ is a normalizing constant.

*Proof.* Let σ and τ be any two SOPS configurations with σ ≠ τ such that Pr (σ, τ) > 0, implying that τ can be reached from σ by a single move of some particle *P*. Suppose *P* has *n* neighbors in σ and has *n*′ in τ. We must show the detailed balance condition holds with respect to the transition probabilities

Pr (σ,τ)π(σ)=Pr (τ,σ)π(τ)

The algorithms in (*26*, *29*) were designed using the Metropolis-Hastings algorithm (*28*), which specifies transition probabilities Pr (σ, τ) = min {π(τ)/π(σ),1} to capture the ratio between stationary weights of the current and proposed configurations. So, we have that π(τ)/π(σ) = λ^*n*^′^−*n*^. It is then easy to see that this ratio is unchanged by the modified transition probabilities where Pr (σ, τ) = λ^−*n*^ and Pr (τ, σ) = λ^−*n*^′^^, and thus, detailed balance is satisfied

$$\frac{\text{Pr}\;(\sigma ,\tau )}{\text{Pr}\;(\tau ,\sigma )}=\frac{\lambda^{-n}}{\lambda^{-n\prime }}=\lambda^{n\prime -n}=\frac{\pi (\tau )}{\pi (\sigma )}$$

Therefore, since π satisfies detailed balance and ℳ_AGG_ is an ergodic finite Markov chain, we conclude that π is the unique stationary distribution of ℳ_AGG_.

We conclude by outlining the proof of Theorem 2 that shows that ℳ_AGG_ achieves aggregation when λ is large enough and dispersion when λ is close to one. Our proof is a series of information-theoretic arguments about the stationary distribution π. We use ideas similar to Peierls arguments, which are often used in statistical physics to study phase changes in behavior space for infinite systems (*56*). In (*29*), it was shown that, for finite systems, particles of two different colors could either separate into monochromatic clusters or integrate, indifferent to color. This separation algorithm can be applied to the setting where a bounded region of the lattice is completely filled with particles that move by “swapping” places with their neighbors. By viewing particles of one color as “empty space” and particles of the other color as our particles of interest, the swap moves in the separation algorithm correspond to particle moves within a bounded area. These are precisely the moves used in our aggregation algorithm, where separation corresponds to aggregation and integration corresponds to dispersion. Thus, it is straightforward to leverage the arguments for separation and integration in (*29*) to show aggregation and dispersion in a bounded region.

For large enough bias λ, we prove that aggregation occurs with high probability as follows. Using techniques introduced in (*57*), we define a map from any configuration without an aggregate to a configuration with an aggregate by (i) choosing some scattered particles in a systematic way and (ii) rearranging them as an aggregate in a carefully chosen location. We then show that no aggregate configuration has too many preimages under this map because of the careful way we remove scattered particles. On the other hand, we show that applying this map to a dispersed configuration leads to a large increase in its stationary probability. Provided λ is large enough that the probability gain outweighs the number of preimages, these two facts imply that aggregated configurations are much more likely to occur in the stationary distribution than dispersed ones. More formally, the above argument shows that the stationary probability of being in a dispersed configuration is at most ${\left(c_{1}/\lambda \right)}^{c_{2}\sqrt{N}}$, where *c*_1_, *c*_2_ > 0 are constants that depend on the map described above. Thus, provided λ is large enough, this probability of being in a dispersed configuration is very small, proving that aggregation is achieved with high probability.

When the bias λ is close to one, we can prove that dispersion occurs with high probability. We show that there exist polynomially many events such that if aggregation occurs, then at least one of these events must also occur. These events correspond to certain regularly shaped subregions of the lattice being almost entirely occupied by particles. We then use a Chernoff-type bound to show that each of these events is exponentially unlikely when λ is close to one. This implies that the stationary probability for aggregated configurations is at most the sum of polynomially many terms that are each exponentially small, so dispersion must occur with high probability for this range of λ.

### BOBbot design

The BOBbot mechanical design was developed in SolidWorks, and its skeleton was three-dimensionally printed in ABS (acrylonitrile butadiene styrene) plastic by a Stratasys uPrint SE Plus printer at a layer resolution of 0.254 mm and sparse density (fig. S1). Each BOBbot contains a lithium ion polymer battery (Adafruit Industries) that is equipped with Qi wireless charging for recharging between experiments (Adafruit Industries). The brushbot design is implemented using an ERM (BestTong) for vibrations and two Pienoy dog toothbrush heads as feet, yielding noisy circular trajectories (movie S2). The BOBbot’s motor circuitry was assembled on a printed circuit board (PCB) designed in Eagle CAD (fig. S2). The PCBs were printed at the Georgia Tech Interdisciplinary Design Commons makerspace and outsourced from JLCPCB. This circuitry is switched and modulated by a phototransistor (Adafruit Industries), which acts as a proportional controller for motor speed. Grade N42 neodymium magnets (K&J Magnetics) are housed in the BOBbot chassis for inter-robot attraction and can be swapped for magnets of different strengths to modulate the BOBbots’ cohesion. A complete list of BOBbot components can be found in table S1.

To achieve stress sensing, each BOBbot is equipped with four triggers that mechanically deform and close the circuit upon collisions to sense the locally exerted stress (Fig. 6B and fig. S2). These triggers are positioned radially in front of the permanent magnets in the chassis. The stress sensors function such that a robot decreases its motor speed for sufficiently large stress (Fig. 6C). The analog circuit is designed to reduce the motor’s current in a manner proportional to the total number of contacts, starting with roughly 70% reduction for a single triggered sensor (Fig. 6C, top). When multiple sensors are triggered, a BOBbot’s speed is practically negligible.

### Simulations

To simulate the SOPS, we execute the algorithm on a hexagonal lattice. The size of the lattice in Fig. 1 is chosen to be sufficiently large so that boundary effects are mitigated. The size of the lattice for Fig. 4 is chosen to match the area density and the number of agents in the physical evolution and algorithm. To determine the constant *k*_0_ in the aggregation metric $\text{AG}G_{\mathrm{MC}}=N_{\mathrm{MC}}/(k_{0}P_{\mathrm{MC}}\sqrt{N})$, we consider a hexagon with area $N_{\mathrm{MC}}=\frac{\sqrt{3}}{4}\ell^{2}\cdot 6$ and perimeter *P_MC_* = 6ℓ, setting *k*_0_ so that AGG*_MC_* = 1. This yields $k_{0}=1/\sqrt{8\sqrt{3}}$.

Beyond the information described in the main text, the DEM simulations faithfully represent the spherical loose magnets with exponentially decaying force housed in each BOBbot’s chassis slots, resulting in patchy magnetic interaction as the magnets move freely in their slots. Attraction between two simulated BOBbots is calculated on the basis of these magnetic spheres’ strength and the minimum physical separation between any interacting pair, which depends on the relative position and orientation of the two BOBbots.

To calibrate our DEM simulations, we measure the BOBbots’ physical parameters and use these values for the simulated BOBbots (Table 1). Most parameters such as the mass and dimensions of each BOBbot are directly measured. For others, we use a series of experiments designed to isolate individual parameters. For instance, to avoid possible system errors such as in-plane friction when measuring the magnetic force, we measured the minimum force needed to overwhelm the magnetic force in vertical direction (fig. S4). Other indirect measurements involve the translational and rotational drag (figs. S5 and S6). The key ingredient in these experiments is to use a known force (Earth’s gravity) to calibrate these intricate forces. Details can be found in section S2.

**Table 1:** List of parameters used in physical simulations.

	**Description**	**Experiment**	**Simulation**
*m*	BOBbot mass	0.060 kg	0.060 kg
*R*_0_	BOBbot radius	0.030 m	0.030 m
*I*	BOBbot moment of inertia	2.7 × 10^−5^ kg·m^2^	2.7 × 10^−5^ kg·m^2^
*R_C_*	Radius of the regular circular motion	25 ± 5 mm	25 mm
*R*_*B*_0__	Radius of the magnetic bead	2.3 mm	2.0 mm
*R_S_*	Thickness of the magnet cavity shell	2.0 mm	2.0 mm
*R_B_*	Effective radius of the magnetic bead	4.3 mm	4.0 mm
*v*_0_	Saturated speed	48.4 ± 20.2 mm/s	60.0 mm/s
ω_0_	Saturated angular velocity of the orbit	1.94 ± 0.81 rad/s	2.4 rad/s
*F_D_*	Translational drive	0.07 N	0.06 N
τ*_D_*	Rotational drive (torque)	5 × 10^−4^ N·m	5.5 × 10^−4^ N·m
η	Translational drag coefficient	∼1 kg/s	1.0 kg/s
η_φ_	Rotational drag coefficient	≤3 × 10^−4^ N·m·s	2.3 × 10^−4^ N·m·s
*F*_*M*0_	Magnetic force on contact	3–35 gf	3–35 gf
*d*_0_	Magnetic force decay length	1.5 mm	1.5 mm
μ	Bot-bot friction coefficient	0.143	0.143
μ*_W_*	Bot-wall friction coefficient		0.143

The DEM simulations use the Euler-Maruyama method with a time step of 1 ms to integrate the following Newton equations

$$m\ddot{\vec{r}}=F_{D}\hat{u}-\eta \overset{\cdot }{\vec{r}}+{\vec{F}}_{\text{env}}(\vec{r},\varphi )+\vec{\xi }(t)$$

$$I\ddot{\varphi }=\tau_{D}-\eta_{\varphi }\overset{\cdot }{\varphi }+\tau_{\text{env}}(\vec{r},\varphi )+\xi_{\varphi }(t)$$

As the agents are in the overdamped regime where $|m\ddot{\vec{r}}|\ll \eta |\overset{\cdot }{\vec{r}}|$, the Newton equations are equivalent to the Langevin equations for active Brownian particles by taking the limit *m*, *I* → 0.

$$\overset{\cdot }{\vec{r}}=v_{0}\hat{u}+{\vec{F}}_{\text{env}}(\vec{r},\varphi )/\eta +\vec{\xi }(t)/\eta$$

$$\overset{\cdot }{\varphi }=\omega_{0}+\tau_{\text{env}}(\vec{r},\varphi )/\eta_{\varphi }+\xi_{\varphi }(t)/\eta_{\varphi }$$

As we see from the reduced equations, in the steady state, a BOBbot will perform a circular motion with a saturated speed *v*_0_ = *F_D_*/η and a frequency of ω_0_ = τ*_D_*/η_φ_. This suggests that we can control a BOBbot’s speed *v*_0_ by changing its motor vibration strength, varying *F_D_*.

The initial placement of the BOBbots is achieved by greedy rejection sampling, sequentially placing BOBbots in random positions that do not overlap with the previously placed BOBbots. A cell list search method is used to speed up the simulation’s computation by subdividing the simulated arena into square cells so that, when integrating forces for a given BOBbot, we only consider interactions with BOBbots from the same or adjacent cells. The size of the cells is chosen such that the relative error caused by this approximation is within 10^−3^.

## SUPPLEMENTARY MATERIALS

Supplementary material for this article is available at http://advances.sciencemag.org/cgi/content/full/7/17/eabe8494/DC1
